# A pilot prospective study on closed loop controlled ventilation and oxygenation in ventilated children during the weaning phase

**DOI:** 10.1186/cc11343

**Published:** 2012-05-16

**Authors:** Philippe Jouvet, Allen Eddington, Valérie Payen, Alice Bordessoule, Guillaume Emeriaud, Ricardo Lopez Gasco, Marc Wysocki

**Affiliations:** 1 Pediatric intensive Care Unit, Sainte-Justine Hospital, 3175 Chemin Côte Sainte Catherine, Montreal H3T 1C5, Canada; 2 Research Center, Sainte-Justine Hospital, 3175 Chemin Côte Sainte Catherine, Montreal H3T 1C5, Canada; 3 R&D Department, Hamilton Medical, 4 Engelstrasse, Wädenswil, 8820 Switzerland

## Abstract

**Introduction:**

The present study is a pilot prospective safety evaluation of a new closed loop computerised protocol on ventilation and oxygenation in stable, spontaneously breathing children weighing more than 7 kg, during the weaning phase of mechanical ventilation.

**Methods:**

Mechanically ventilated children ready to start the weaning process were ventilated for five periods of 60 minutes in the following order: pressure support ventilation, adaptive support ventilation (ASV), ASV plus a ventilation controller (ASV-CO_2_), ASV-CO_2 _plus an oxygenation controller (ASV-CO_2_-O_2_) and pressure support ventilation again. Based on breath-by-breath analysis, the percentage of time with normal ventilation as defined by a respiratory rate between 10 and 40 breaths/minute, tidal volume > 5 ml/kg predicted body weight and end-tidal CO_2 _between 25 and 55 mmHg was determined. The number of manipulations and changes on the ventilator were also recorded.

**Results:**

Fifteen children, median aged 45 months, were investigated. No adverse event and no premature protocol termination were reported. ASV-CO_2 _and ASV-CO_2_-O_2 _kept the patients within normal ventilation for, respectively, 94% (91 to 96%) and 94% (87 to 96%) of the time. The tidal volume, respiratory rate, peak inspiratory airway pressure and minute ventilation were equivalent for all modalities, although there were more automatic setting changes in ASV-CO_2 _and ASV-CO_2_-O_2_. Positive end-expiratory pressure modifications by ASV-CO_2_-O_2 _require further investigation.

**Conclusion:**

Over the short study period and in this specific population, ASV-CO_2 _and ASV-CO_2_-O_2 _were safe and kept the patient under normal ventilation most of the time. Further research is needed, especially for positive end-expiratory pressure modifications by ASV-CO_2_-O_2_.

**Trial registration:**

ClinicalTrials.gov: NCT01095406

## Introduction

The worldwide increase in patient complexity, more concern for quality and safety, and a shortage of resources are the challenging terms of the equation many physicians and respiratory therapists have to face nowadays [1-3]. Mechanical ventilation is one of the life-saving techniques used at the bedside that is also associated with complications, including ventilator-induced lung injury and ventilator-associated pneumonia [4,5]. To decrease the duration of mechanical ventilation, there is evidence suggesting that the use of a written protocol is helpful [6]. Writing and reading protocols are time consuming, resulting in fluctuation in protocol implementation and compliance; instructions cannot be explicit enough, resulting in variable interpretation of the protocol; and they are specific to one organisation, making transfer to another institution difficult. To overcome these limiting factors, protocols have been computerised and there is convincing evidence showing that computerised protocols may help to manage and wean adult patients [7,8] and children with severe lung diseases [9,10].

A new computerised protocol with closed loop control of ventilation and oxygenation (IntelliVent^®^; Hamilton Medical AG, Bonaduz, Switzerland) has recently become available and has been investigated in adult patients [11]. The present study is a pilot prospective evaluation of this new closed loop computerised protocol in stable mechanically ventilated children during the weaning phase.

## Materials and methods

The study - which was approved by the Sainte Justine Hospital ethical committee (Montreal, Canada) and by Health Canada and was registered on clinicaltrials.gov (NCT01095406) - was realised between January 2010 and January 2011 in the paediatric ICU of the Sainte Justine Hospital, Montreal, Canada. Signed informed consent was obtained from legal representatives as well as consent for publication of this manuscript and accompanying images.

### Patients

During the study period, all consecutive admitted critically ill children under mechanical ventilation for at least 12 hours, younger than 18 years old, having predicted body weight (PBW) ≥ 3 kg and body mass index < 40 kg/m^2 ^were screened daily for the study 5 days a week (Monday to Friday). PBW was the weight at the 50th percentile for age and sex from the National Center of Health Statistics growth chart.

The study was in essence a pilot investigation and was designed primarily to evaluate the safety of the new computerised protocol. Children were therefore included if they fulfilled the following criteria: able to breathe spontaneously; ventilated with proximal airway plateau pressure ≤ 25 cmH_2_O, positive end-expiratory pressure (PEEP) ≤ 8 cmH_2_O, with fraction of inspired oxygen (FiO_2_) ≤ 60% to keep oxygen saturation from pulse oxymetry (SpO_2_) ≥ 95%, and arterial partial pressure of CO_2 _< 70 mmHg on the last arterial blood gas; endotracheal tube leakage < 20% of the inspired tidal volume (VT), because the new computerised protocol requires correct information on VT; arterial partial pressure of CO_2 _versus partial pressure in end-tidal CO_2 _(PEtCO_2_) difference ≤ 7 mmHg; or not receiving muscle relaxant, vasopressor or inotropic medication (except dopamine < 5 μg/kg/minute).

Children with a previous history of mechanical ventilation (more than 1 month) or tracheotomy, severe neuromuscular disease, cyanotic congenital heart disease and primary pulmonary hypertension were excluded, as well as brain-death children and children receiving palliative care.

An analysis was planned after the inclusion of five patients (run-in phase) because there was no clinical experience of this mode of ventilation. The analysis of the run-in phase showed that children with PBW < 7 kg had an increase in minute ventilation (MV) because of technical reasons (high apparatus dead space with the S1 device due to the proximal flow sensor + mainstream CO_2 _analyser in series). The investigators decided to only include children with PBW > 7 kg. The four patients under 7 kg PBW and already included in the run-in phase were not analysed, although they went through the study without adverse effects (see Safety evaluation below).

### Study design

**Figure 1:**
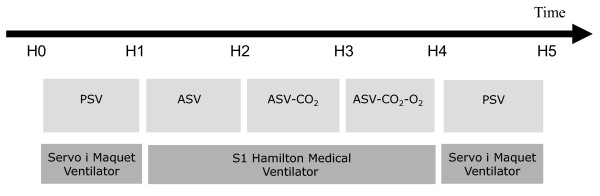
**Study protocol**. Included patients were prospectively enrolled in a sequential study during which they received five consecutive 1-hour periods of ventilation. PSV, pressure support mode; ASV, adaptive support ventilation mode; ASV-CO_2_, ASV and CO_2 _controller; ASV-CO_2_-O_2_, ASV-CO_2 _and oxygen controller.

All included patients were prospectively enrolled in a sequential nonrandomised study during which they received five consecutive 60-minute periods of ventilation (Figure 1): pressure support ventilation (PSV) using the ventilator already connected to the patient (Servo-*i*; Maquet Gmbh & Co. KG, Rastatt, Germany) with the level of inspiratory pressure (P_insp_) set by the attending intensivist prior to the inclusion (PSV_before); adaptive support ventilation (ASV) using a functional S1 prototype ventilator (Hamilton Medical AG); ASV with the CO_2 _controller activated (ASV-CO_2_); ASV with the CO_2 _and the O_2 _controller activated (ASV-CO_2_-O_2_); and PSV using the Servo-*i *ventilator at the initial level of support (PSV_after).

Throughout the ventilation period with the S1 device and for safety reasons, a trained physician remained at the bedside.

The PSV mode was given with the servo-*i *ventilator because the S1 device had only the new computerised protocol and ASV inside. The ventilation modes were not randomised as we had to ventilate the children in PSV before and at the end of the study, and randomising the modes would require changing the ventilator in a randomised order as well, which was not allowed for safety reasons.

### Ventilatory modalities

ASV is a ventilatory modality commonly used in adults [11,12] where, for a given MV set by the user according to the clinical conditions and arterial blood gases, the ventilator automatically selects an optimal combination of respiratory rate (RR) and VT based on the breath-by-breath estimation of the expiratory time constant. The level of support (mandatory rate and P_insp_) is automatically adjusted on a breath-by-breath basis to keep the patient as close as possible to the optimal RR and VT. If the patient breathes spontaneously, this mode works as a volume target-pressure regulated mode with a user-set MV guarantee. If not, the ASV mode works as a pressure control mode with a prescribed MV. In ASV, the MV is the parameter that is controlled and set by the user. To further control the VT (and more specifically to reduce the VT) or to limit P_insp_, the user must decrease the MV and the maximum P_insp _allowed (pressure limitation). At the bench, in doing so it has been recently shown that ASV was able to generate lower VT and P_insp _compared with the ARDSnetwork recommendations [13]. However, in some clinical studies when the maximum allowed pressure is permissive (60 cmH_2_O), high VT has been reported with ASV [14]. In our study, MV in ASV was set to match the MV measured during PSV_before. PEEP and FiO_2 _were set as during PSV_before. The pressure limitation was set to limit the P_insp _to 25 cmH_2_O.

**Figure 2:**
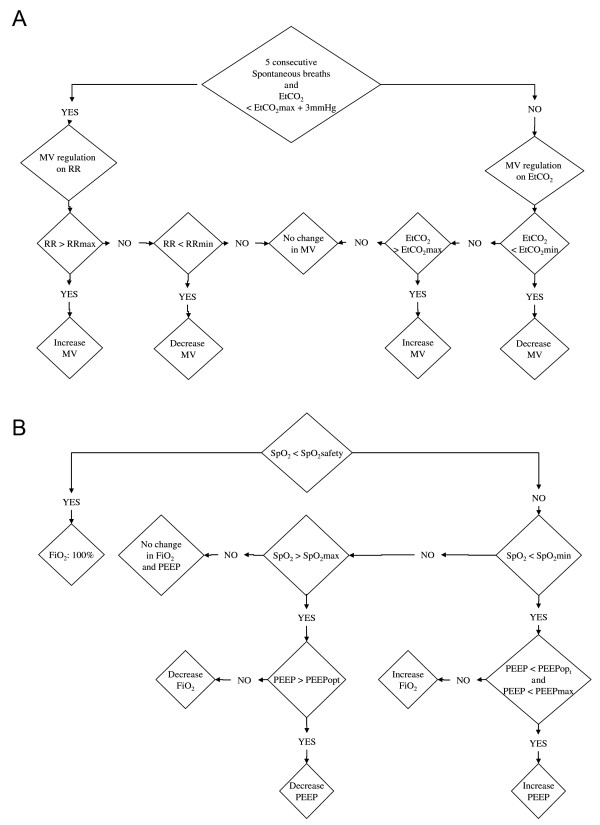
**Functional algorithm of the ventilation and oxygen controller**. **(A) **Ventilation controller. The partial pressure in end-tidal CO_2 _(PEtCO_2_) values are given with the quality index and derived from the mainstream CO_2 _sensor with proprietary algorithms as a surrogate of arterial partial pressure of CO_2_. PEtCO_2_min and PEtCO_2_max are adjustable by the user and depend on the patient's severity estimated by the level of inspiratory pressure; that is, the higher the inspiratory pressure and the more permissive the PEtCO_2 _limits. As an example and by default for an inspiratory pressure of 10 cmH_2_O, PEtCO_2_min is 35 mmHg and PEtCO_2_max is 41 mmHg. **(B) **Oxygen controller. The oxygen saturation from pulse oxymetry (SpO_2_) limits (SpO_2_safety and SpO_2_min) are adjustable by the user and depending on the patient's severity estimated by the positive end-expiratory pressure (PEEP) level; that is, the higher the PEEP level and the more permissive the SpO_2 _limits. As an example and by default for a PEEP level of 5 cmH_2_O, SpO_2_safety is 88%, SpO_2_min is 93% and SPO_2_max is 98%. The PEEPopt is defined according to a PEEP-fraction of inspired oxygen (FiO_2_) table and PEEPmax set by the user. The patient SpO_2 _is provided with a quality index and is derived from the pulse oxymeter with proprietary algorithms for artefact and motion rejections. MV, minute ventilation; RR, respiratory rate.

ASV-CO_2 _is an evolution of ASV where MV is not set by the user but is automatically adjusted to keep the patient within ranges of RR and PEtCO_2_, captured at the proximal airway using the S1 flow sensor (PN 279331, single-use flow sensor linear between -120 and 120 l/minute with ± 5% error of measure; Hamilton Medical AG) and a mainstream CO_2 _analyser (Capnostat 5, mainstream sensor with accuracy ± 5% for PEtCO_2 _between 41 and 70 mmHg, dead space 5 ml; Hamilton Medical AG). The target range of RR is defined by a lower limit based on the Otis concept of optimal RR to minimise the work of breathing [15], and an upper limit depending on the actual MV; the higher the MV, the higher the upper RR limit. P_insp _increases when the patient RR is above the upper RR limit; P_insp _decreases when the patient RR is below the lower RR limit. Filtered PEtCO_2 _is present in the background to keep the patient below the upper PEtCO_2 _acceptable value (Figure 2). Ventilation changes are made on a breath-by-breath basis.

ASV-CO_2_-O_2 _includes the above-described ASV-CO_2 _regulation plus an automatic adjustment of PEEP and FiO_2 _based on SpO_2 _monitoring included in the ventilator. SpO_2 _is obtained from a finger probe using standard technology, plus proprietary filtering and quality assessment before entering the algorithm: when SpO_2 _is below a user-adjustable value, FiO_2 _or PEEP is increased (Figure 2). These ventilation changes are made on a breath-by-breath basis. The choice between FiO_2 _and PEEP increase is based on the ARDSnetwork tables [16] with user inputs regarding maximal and minimal PEEP values. The FiO_2 _controllers and the PEEP controller can be activated or deactivated independently. In the present study both controllers were activated, but according to local policies regarding PEEP settings the minimal PEEP and maximal PEEP were respectively set at 5 and 10 cmH_2_O.

The investigators remained at the bedside during the entire duration of the study and were allowed to switch back to manual adjustment of the settings. As for any mode of ventilation, the user has to set alarms for VT, for airway pressure as well as for monitoring parameters such as SpO_2 _and PEtCO_2_.

### Data collection

Patient characteristics including demographic data, diagnosis, severity scores, clinical data at inclusion and outcomes were collected from the charts. When connected to the S1 device, monitoring values (including SpO_2_, PEtCO_2 _and mechanical ventilation monitoring), settings and alarms were recorded on a breath-by-breath basis using a dedicated data-logging system attached to the ventilator. Minute-to-minute data from the Servo-*i *were collected using a compact flash reader connected to the device.

Breath-by-breath data were analysed *ex vivo *to calculate the number of normal breaths as defined by 10 breaths/minute < RR < 40 breaths/minute, VT > 5 ml/kg PBW and 25 mmHg < PEtCO_2 _< 55 mmHg. The primary end point of the study was the percentage of time spent with normal ventilation, defined as the number of normal breaths out of the total number of breaths collected. During PSV_before and PSV_after, PEtCO_2 _was dropped from the definition as it was not recorded with the Servo-*i *ventilator.

The number of ventilation setting changes was compared between the five consecutive 60-minute periods of ventilation. A ventilation setting change corresponded to a modification of PEEP or FiO_2 _in any ventilation mode, a change of P_insp _in PSV or a change of minute volume in ASV, ASV-CO_2 _or ASV-CO_2_-O_2_.

### Safety evaluation

Safety was evaluated by recording adverse events as defined by ISO 14155 standards (ISO 14971:2007, Medical devices -- Application of risk management to medical devices) and by the number of switch back to PSV or to controlled ventilation by the intensivist present at the bedside, whatever the reason. The protocol was terminated and the child ventilated with the previous conventional ventilation if a sustained change was demonstrated in any of the following: decrease in SpO_2 _< 92% requiring increase in FIO_2 _> 60%; increase in PEtCO_2 _above the value before connection with the S1 device, requiring an increase of positive P_insp _> 25 cmH_2_O above PEEP; increase in heart rate > 180 beats/minute for 15 minutes; increase in RR > 60 breaths/minute for 15 minutes; or uncontrolled agitation.

### Sample size

There were no previously existing data on the percentage of time spent with normal ventilation in children during mechanical ventilation. A similar study in adults observed that patients in PSV mode spent 66 ± 23% in the normal ventilation range compared with 93 ± 8% of patients ventilated with a closed loop computerised protocol [17]. From this study [17], we calculated that 16 patients would be required to find a statistical difference for such a difference with an α risk of 0.05 and a β risk of 0.9.

### Statistical analysis

As the parameters studied did not have a normal distribution, data are shown as the median with 25% and 75% interquartile ranges. Medians were compared using a Kruskal-Wallis one-way analysis of variance on ranks and paired tests when *P *< 0.05 using Sigma Stats for Windows version 3.5 (Systat Software, Inc., Richmond, CA, US).

## Results

During the study period, 196 patients were screened and 15 patients with PBW > 7 kg were included. These 15 patients were ventilated for a median of 55 hours before inclusion (Table 1). One patient (Patient 12, Table 1) did not undergo the ventilation periods with the S1 device due to the use of a neonatal flow sensor by mistake, which did not allow ventilating in the ASV mode, and was therefore excluded from analysis. The remaining 14 patients went through the study without side effects, without switching to PSV or controlled ventilation, or without early protocol termination when they were ventilated with the S1 ventilator.

**Table 1:** Clinical description of the patients at inclusion

Patient	Age (months)	PBW (kg)	PIM2 (%)	PELOD	Main diagnosis	Data at inclusion					
						
						MV duration (hours)	Ppeak (cmH_2_O)	SpO_2 _(%)	**FiO**_**2**_	PEEP (cmH_2_O)	PaCO_2 _(mmHg)
1	36	12	0	12	Rhabdomyosarcoma	21	15	100	0.25	5	36
2	195	57	0	2	Scoliosis-encephalopathy	26	13	98	0.25	5	34
3	93	27	3	2	Polytraumatism	240	15	98	0.30	5	40
4	28	14	2	3	Liver transplantation	72	25	98	0.40	7	46
5	59	17	0	1	Cranio-facial surgery	56	17	98	0.25	5	47
6	22	14	3	1	Laryngitis	44	15	96	0.35	5	38
7	187	38	0	2	Congenital scoliosis	55	21	99	0.25	5	39
8	29	10	3	2	Septic shock	203	26	95	0.40	5	48
9	32	14	1	3	Encephalitis	46	15	100	0.30	5	38
10	54	16	0	1	Thyreoglosse cyst	31	15	99	0.45	5	39
11	9	7	3	1	Severe laryngitis	41	18	98	0.35	5	41
12	62	18	1	11	Pharyngeal abscess	38	17	98	0.21	5	37
13	45	15	13	1	Aspiration-encephalopathy	235	11	98	0.30	5	59
14	16	10	3	14	Encephalitis	66	15	100	0.25	5	29
15	165	49	2	12	Encephalopathy	75	15	98	0.30	5	40
Median	45	15	2	2		55	15	98	0.30	5	39
IQR25	29	13	0	1		40	15	98	0.25	5	38
IQR75	78	23	3	7		73	18	99	0.35	5	43

PBW, body weight; PIM2, Paediatric Index of Mortality (percentage of risk of death) [29]; PELOD, Paediatric Logistic Organ Dysfunction score [30]; MV, mechanical ventilation; Ppeak, peak pressure at the proximal airway; SpO_2_, pulse oxygen saturation; FiO_2_, inspired oxygen fraction; PEEP, positive end-expiratory pressure; PaCO_2_, arterial partial pressure of CO_2_; IQR25 and IQR75, 25% and 75% interquartile range.

Compared with PSV_before (93% (79 to 95%)) and PSV_after (96% (83 to 100%)), ASV, ASV-CO_2 _and ASV-CO_2_-O_2 _kept the patients within normal ventilation for a similar percentage of time (respectively: 93% (80 to 95%), 94% (91 to 96%) and 94% (87 to 96%)) (Table 2).

**Table 2:** Time spent with normal ventilation, ventilation pattern, peak airway pressure, PEtCO_2 _and SpO_2_

	PSV_before	ASV	**ASV-CO**_**2**_	**ASV-CO**_**2**_**-O**_**2**_	PSV_after	*P *value
Normal ventilation (% of the recording time)	93 (82 to 95)	94 (82 to 96)	95 (92 to 96)	95 (89 to 96)	97 (85 to 100)	NS
Number of ventilation setting changes/patient	0 (0 to 0)	0 (0 to 0)	78 (58 to 119)	81 (35 to 250)	0 (0 to 0)	< 0.001*
Respiratory rate (breaths/minute)	17 (14 to 21)	21 (18 to 24)	23 (19 to 25)	22 (18 to 27)	20 (16 to 26)	NS
Tidal volume (ml/kg)	8.3 (7.1 to 9.3)	8.7 (7.7 to 9.7)	8.9 (8.2 to 9.7)	8.4 (7.8 to 9.7)	7.9 (6.7 to 8.9)	NS
Paw-peak (cmH_2_O)	16 (15 to 18)	15 (14 to 22)	16 (14 to 19)	17 (15 to 21)	15 (14 to 19)	NS
PEtCO_2 _(mmHg)	-	43 (39 to 45)	43 (40 to 45)	43 (39 to 47)	-	NS
SpO_2 _(%)	-	98 (95 to 100)	99 (96 to 100)	98 (95 to 100)	-	NS

Results of time spent with normal ventilation, ventilation pattern, peak airway pressure, partial pressure of end-tidal CO_2 _(PEtCO_2_) and pulse oxygen saturation (SpO_2_) during the five 1-hour periods investigated. Results given as the median with 25% to 75% interquartile range. Normal ventilation is the percentage of time with normal breath as defined by 10 breaths/minute < respiratory rate < 40 breaths/minute, tidal volume > 5 ml/kg predicted body weight and 25 mmHg < PEtCO_2 _< 55 mmHg. PSV, pressure support ventilation; ASV, adaptive support ventilation, ASV-CO_2_, ASV plus the CO_2 _controller; ASV-CO_2_-O_2_, ASV-CO_2 _plus the oxygen controller; Paw-peak, peak proximal airway pressure; NS, *P *> 0.05. *P < 0.001 for ASV versus ASV-CO_2 _and for ASV versus ASV-CO_2_-O_2_.

**Figure 3:**
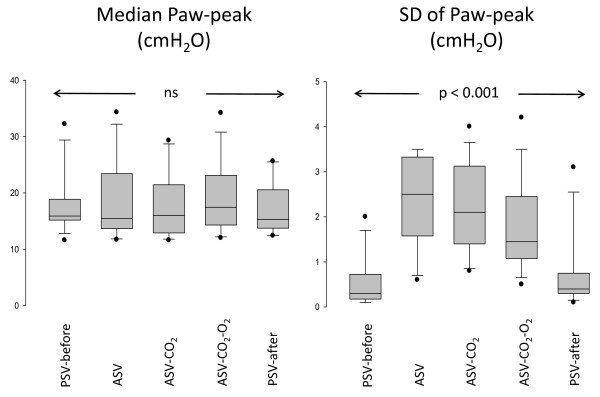
**Peak airway pressure of children during the 1-hour periods of mechanical ventilation**. Peak inspiratory airway pressure (Paw-peak; median and standard deviation (SD)) of the 14 children included during the five 1-hour periods of mechanical ventilation. The median values were not statistically different, but the SDs of individual breath-by-breath values (right panel) were significantly higher, suggesting more variability in adaptive support ventilation (ASV), ASV and CO_2 _controller (ASV-CO_2_) and ASV-CO_2 _and oxygen controller (ASV-CO_2_-O_2_) as compared with pressure support ventilation (PSV).

The VT, RR, peak inspiratory airway pressure (Paw-peak) and MV were equivalent with all modalities. PEtCO_2 _and SpO_2 _were also equivalent between ASV, ASV-CO_2 _and ASV-CO_2_-O_2 _(Table 2). The standard deviation to estimate the variability of Paw-peak was much higher with ASV, ASV-CO_2 _and ASV-CO_2_-O_2 _as compared with PSV (Figure 3). As compared with ASV, there were significantly more ventilator setting adjustments (automatic) in ASV-CO_2 _and ASV-CO_2_-O_2 _(77 (57 to 120) per patient and 80 (32 to 272) per patient, respectively) (Table 2).

In four patients, during the 60-minute period with ASV-CO_2_-O_2_, an automatic increase of PEEP from 5 cmH_2_O to 7 to 9 cmH_2_O was observed due to a transient decrease in SpO_2_. The physician manually returned the PEEP to 5 cmH_2_O because it was not in his practice to increase PEEP in this situation.

The median total duration on the ventilator was 97 hours (55 to 207 hours) and the length of stay in the ICU was 5 days (4 to 13 days). At 28 days after the inclusion, all patients were alive, none was on mechanical ventilation and five patients remained in the hospital.

## Discussion

ASV alone or combined with the automatic adjustment of minute volume was safe and provided a similar breath pattern as compared with PSV. This was achieved, however, with more ventilator automatic adjustments and more variability in Paw-peak as compared with ASV and PSV. The apparatus dead space with the S1 device due to the proximal flow sensor + mainstream CO_2 _analyser in series restrained the ventilation to children with body weight > 7 kg. As the patients were in a stable situation and not hypoxemic, little information was obtained regarding the oxygenation controller. In four patients, however, an automatic increase in PEEP was observed that was not considered usual practice by the physician in charge.

The present pilot study has several limitations that need to be addressed before raising some conclusions. First, comparing ASV, ASV-CO_2 _and ASV-CO_2_-O_2 _from the S1 device with PSV given with another ventilator is arguable. Even if the aim of the study was not to compare the performance of each mode in supporting the patient, this may indeed impact on the primary endpoint - that is, the time spent with normal ventilation, which was defined based on the RR, VT and PEtCO_2_. Furthermore, the data were acquired differently with the S1 device (breath by breath) as compared with the Maquet ventilator (minute by minute, equivalent to a filtering). PEtCO_2 _was also not part of the definition of normal breathing in PSV, and PEtCO_2 _is known to show high fluctuation on both sides in spontaneously breathing patients [18]. Whether minute-by-minute filtering and not having PEtCO_2 _in PSV are overestimating the time in normal ventilation during PSV is questionable. As the S1 ventilator did not have the PSV mode, however, the study had to be carried out with the methodology described above.

Second, the different modes of ventilation were given sequentially and without randomisation. The total duration of the study was 5 hours and very obviously the patient's conditions may change over such a long period. However, the patient's respiratory conditions in PSV_after as compared with PSV_before were not different (Table 2).

Third, the study was finally underpowered (14 patients analysed as compared with the 16 patients required by the power calculation). This was because of four patients weighing < 7 kg and not being analysed. In one patient the new computerised protocol was also unable to work because of the wrong flow sensor (neonatal flow sensor) being on the device. However, an increased number of patients would probably not achieve statistical significance as the observed time spent in normal ventilation during PSV mode was higher than expected (93% instead of 66% as expected from a previous adult study [17]).

Fourth, the different modes were investigated over a short period of time (60 minutes) and in a very specific subpopulation of stable patients during the weaning phase. It is indeed impossible to estimate the safety of such a closed loop computerised protocol in more critically ill patients. The clinical impact of such a mode of ventilation is also not addressed by the present study. These studies will be the next steps.

Finally, the PEEP controller - which is probably the most critical in terms of safety - was set to very conservative working conditions (minimal PEEP 5 cmH_2_O and maximal PEEP 10 cmH_2_O). In four patients an increase of PEEP from 5 cmH_2_O to 7 to 9 cmH_2_O was observed due to a transient decrease in SpO_2_. The physician manually returned the PEEP to 5 cmH_2_O because it was not in his practice to increase PEEP in this situation. The present study is therefore not able to raise any conclusions regarding the PEEP closed loop computerised protocol, and further data are needed on the optimal PEEP during the weaning phase.

The present pilot study is therefore exploratory and can only indicate that in this stable population, over a short period of time (60 minutes) and within the experimental conditions (PEEP limited and manually controlled), ASV-CO_2 _and ASV-CO_2_-O_2 _were safe. Additional studies are definitely required before raising any conclusion on safety or effectiveness during longer period of ventilation and in patients with more critical conditions.

Interesting observations can be raised, however, from the present study. First, in babies with body weight < 7 kg, the apparatus dead space (proximal flow sensor in series with the PEtCO_2 _sensor) was relatively high, inducing an increase in MV as compared with PSV. As already shown in adult patients, the dead-space-related increase in MV and subsequent changes in the patient's optimal RR and VT combination is hardly predictable [19]. As most of the children admitted to the paediatric ICU weigh < 7 kg [20], it is critical to reduce the apparatus dead space as much as possible before using such a computerised protocol.

Second, more ventilator automatic adjustments and more variability in Paw-peak were observed in ASV-CO_2 _and ASV-CO_2_-O_2 _as compared with ASV and PSV with ultimately equivalent PEtCO_2_. This is most probably due to the patients' subclinical changing conditions that are taken into account by ASV-CO_2 _and not with ASV or PSV. More variable ventilation may have an impact on lung recruitment, as suggested in atelectatic animal models [21,22], and may have an impact on patient comfort, as suggested by studies on variable modes using pressure support [23,24].

Third, although designed for children and adult patients, and already on the market for 10 years, publications are lacking on ASV in children. In the very first publication from Laubscher and coworkers in 1994 [25], some paediatric patients were included but ASV was applied only for a couple of minutes to test the start-up procedure in selecting the VT and RR. To our best knowledge, ASV has never been compared with PSV in the paediatric population. Bearing in mind the abovementioned limitations and publications from adult populations reporting higher VT with ASV when the pressure limitation is high [14], it is worth noting that breath patterns with both modes were not drastically different. Once again, this would deserve additional investigation.

Finally, although not really challenged by the population enrolled in the study, an automatic control of FiO_2 _based on SpO_2 _has been already investigated in neonates [26,27] and in adults [28], with increased time spent with adequate SpO_2 _when compared with usual care. The present study is in line with these previously published results.

## Conclusion

The present pilot prospective study shows the feasibility of using a closed loop computerised protocol on ventilation and oxygenation over a short time period in stable, spontaneously breathing paediatric patients starting the weaning process. Over the study period, children with body weight > 7 kg were kept with normal ventilation most of the time and no safety issues were reported. Future studies should be designed to address safety and effectiveness of such a closed loop computerised protocol as compared with conventional ventilation in children with more challenging respiratory conditions and over a longer period of time.

## Key messages

• A closed loop computerised protocol seems feasible in paediatric ventilated patients during the weaning phase.

• Compared with PSV, the closed loop computerised protocol may keep patients within normal ventilation for a similar percentage of time.

• Compared with PSV, the closed loop computerised protocol seems to generate more airway pressure variability.

• Compared with PSV, the closed loop computerised protocol may adjust the ventilator more often.

## Abbreviations

ASV: adaptive support ventilation; ASV-CO_2_: adaptive support ventilation with computerised protocol on ventilation; ASV-CO_2_-O_2_: adaptive support ventilation with computerised protocol on oxygenation; CO_2_: carbon dioxide; FiO_2_: fraction of inspired oxygen; MV: minute ventilation; O_2_: oxygen; Paw-peak: peak inspiratory airway pressure; PBW: predicted body weight; PEEP: positive end-expiratory pressure; PEtCO_2_: partial pressure in end-tidal CO_2_; P_insp_: inspiratory airway pressure; PSV: pressure support ventilation; RR: respiratory rate; SpO_2_: oxygen saturation from pulse oxymetry; VT: tidal volume.

## Competing interests

Intellivent^® ^and an S1 respirator were provided for research purposes by Hamilton Medical. PJ was invited twice to present the results of the clinical research on IntelliVent^® ^at international meetings organised by Hamilton Medical without any honoraria. PJ also has a respirator Servo-*i *and an Evita XL, provided for research purposes by Maquet Medical (Solna, Sweden) and Drager Medical (Lübeck, Germany), respectively. PJ receives a research salary from the Fonds de Recherche du Québec - Santé in respiratory critical care. PJ's research on clinical decision support systems is funded by a public grant from the Natural Sciences and Engineering Research Council of Canada. MW was the director of the Research and Development department of Hamilton Medical until the end of 2011. MW co-shares a patent on IntelliVent^® ^(WO/2007/085110 and WO/2007/085108). RLG is a medical research engineer employed by Hamilton Medical. The other authors declare they have no competing interests. These declarations do not mean that the interpretation of data and presentation of information was influenced by these relationships. This is fair research in collaboration with industry to obtain a final product helpful for children.

## Authors' contributions

PJ was the principal investigator of the study, designed the study, was at the bedside for data collection, analysed the results, performed the statistics and wrote the manuscript. AE, VP and AB were co-investigators, screening the patients for inclusion and collecting the data at the bedside. GE was a co-investigator, discussing the study design and reviewing the manuscript. RLG was research engineer for Hamilton Medical and in charge of the logistics of the study, designed and realised the macro for breath-by-breath analysis, and was in charge of storing and securing the data. MW was head of medical research when the study started, was involved in the research plan and in discussing the data and the results of the study, and was involved in finalising the manuscript before the submission. All authors read and approved the final manuscript.
